# Terrestrial arthropods of Steel Creek, Buffalo National River, Arkansas. IV. Asilidae and other Diptera

**DOI:** 10.3897/BDJ.4.e9977

**Published:** 2016-09-07

**Authors:** Michael Joseph Skvarla, Jeffrey K. Barnes, Danielle M. Fisher, Ashley P. G. Dowling

**Affiliations:** ‡ University of Arkansas, Fayetteville, Arkansas, United States of America; § University of Maryland, College Park, Maryland, United States of America

## Abstract

**Background:**

This is the fourth in a series of papers detailing the terrestrial arthropods collected during an intensive survey of a site near Steel Creek campground along the Buffalo National River in Arkansas. The survey was conducted over a period of eight and a half months in 2013 using twelve trap types, including Malaise and canopy traps, Lindgren multifunnel traps, and pan traps.

**New information:**

We provide collection records for 38 species of Asilidae and other Diptera, 7 of which are new state records for Arkansas: (Asilidae) *Lasiopogon opaculus* Loew, 1874; (Lygistorrhinidae) *Lygistorrhina sancthecatharinae* Thompson, 1975; (Stratiomyidae) *Cephalochrysa nigricornis* (Loew, 1866), *Gowdeyana punctifera* (Malloch, 1915), *Sargus decorus* Say, 1824; (Ulidiidae) *Callopistromyia annulipes* Macquart, 1855; and (Xylophagidae) *Rachicerus obscuripennis* Loew, 1863.

## Introduction

The Interior Highlands is mountainous region in the central United States. It has remained exposed for the last 270 million years and has historically acted as a refugium during times of inhospitable climate (Skvarla et al. 2015). Due to its history and geography, the region is a biodiversity hotspot that supports more than 200 endemic species (Allen 1990, Robison and Allen 1995, The Nature Conservancy, Ozarks Ecogegional Assessment Team 2003, Pringle and Witsel 2005, Zollner et al. 2005, Robison et al. 2008, Skvarla et al. 2015). However, compared to similar biodiversity hotspots, such as the Southern Appalachians, the Interior Highlands in general has been poorly surveyed. This paper, which focuses on Asilidae and certain other Diptera, is the fourth in a series that detail a survey at Steel Creek in Northwest Arkansas (see Skvarla et al. 2015 for select Coleoptera, Skvarla et al. 2016b for "Symphyta", and Skvarla et al. 2016a for Heteroptera).

Because this paper covers species from a variety of fly families, we provide the following summaries of each family and lower taxon treated.

Anisopodidae comprises 154 species world wide, including nine that are present in North America, five of which belong to *Sylvicola* (Pape et al. 2011, Pratt and Pratt 1980). Larval *Sylvicola* generally develop in decaying organic matter and may become pestiferous in sewage treatment plants (Pratt and Pratt 1980).

Asilidae, the members of which are commonly known as robber flies, are a diverse family of exclusively predatory flies. More than 7,500 species in approximately 550 genera are known worldwide, of which approximately 1,040 species in 100 genera occur in North America north of Mexico (Pape et al. 2011, Geller-Grimm 2016).

Stratiomyidae include approximately 2700 species arranged in more than 380 genera worldwide; more than 250 species in 41 genera are present in North America north of Mexico ( Woodley 2001). While some species are relatively large-bodied and commonly encountered, such as the black soldier fly, *Hermetia illucens* (Linnaeus, 1758), others are smaller in size and easily overlooked. *Sargus*, *Cephalochrysa*, and *Gowdeyana* are examples of such taxa, and are also relatively depauprate in North America, with 6, 4, and 2 species, respectively (Woodley 2001).

Lygistorrhinidae is an uncommonly collected family of Sciaroidea that is easily recognizable by the generally elongate proboscis and reduced wing venation (Thompson 1975). Of the extant taxa, Lygistorrhina (Probolaeus) Williston, 1896, which is sometimes considered a separate genus, contains nine species and is the only (sub)genus to occur in the New World, with the remaining seven genera and Lygistorrhina (Lygistorrhina) restricted to the Old World (though note that Huerta and Ibañez-Bernal 2008 reported an undescribed L. (Lygisstorrhina) from Mexico) (Papavero 1978, Vockeroth 2009, Fungus gnats online 2015). Only one species, *L. santaecatharinae* Thompson, 1975, is known from North America north of Mexico (Fungus gnats online 2015).

Little is known about lygistorrhinids. Most specimens are collected using passive traps (i.e., Malaise traps) or by sweep netting vegetation and almost nothing is known about their behavior and biology, including the immature stages (Vockeroth 2009).

Ulidiidae, commonly known as picture-wing flies, are distinctive for the striking wing patterning and propensity of some species to wave or flash their wings. The peacock fly, *Callopistromyia annulipes*, is especially noticeable as it often holds its wings above the thorax.

*Rachicerus* Walker, 1854, the most speciose genus of Xylophagidae, contains approximately half of the known species worldwide, and is the only genus of Xylophagidae present in tropical forests (Woodley 2011). It is also the easiest genus of Xylophagidae to identify as it is the only one in which the antennae are composed of ten or more flagellomeres and may be pectinate (Webb 1984). Five species of *Rachicerus* are present in the Nearctic; two are restricted to the coastal forests of the Pacific Northwest and three – *R. fulvicollis* Walker 1854, *R. nitidus* Johnson, 1903, and *R. obscuripennis* Loew, 1863 – are found in the eastern United States and Canada (Webb 1984).

## Sampling methods

### Sampling description

The sampling protocol was covered in detail by Skvarla et al. (2015). The following summary is provided for convenience.

The following traps were maintained within a 4 ha site at Steel Creek, Buffalo National River, Arkansas (see Geographic coverage for a description of the site): five Malaise traps, twenty-five pan traps (five of each color: blue, purple, red, yellow, white) which were randomly arranged under the Malaise traps (one of each color per Malaise trap); fifteen Lindgren multi-funnel traps (five of each color: black, green, purple); four SLAM (Sea, Land, and Air Malaise) traps with top and bottom collectors placed in the canopy; and seventeen pitfall trap sets. Additionally, ten leaf litter samples were collected for Berlese extraction when traps were serviced.

Trap placement began on 8 March 2013 and all traps were set by 13 March 2013, except Lindgren funnels, which were set on 1 April 2013. Traps set earlier than 13 March were reset on that date in order to standardize trap catch between traps. Traps were serviced approximately every two weeks. The last collection of pitfall traps and pan traps occurred on 6 November 2013; Malaise, SLAM, and Lindgren funnel traps were run for an additional month, with the final collection on 4 December 2013. In total, 1311 samples were collected.

RV and marine antifreeze, which contains both propylene glycol and ethanol, was used as the preservative in all traps as it is non-toxic, inexpensive, and preserves specimens reasonably well (Skvarla et al. 2014). Insect escape was impeded by the addition of a squirt of unscented, hypoallergenic dish detergent to the propylene glycol to act as a surfactant. Trap catch was sieved in the field and stored in Whirl-Pak bags in 90% ethanol until sorting.

### Quality control

Samples were coarse-sorted using a Leica MZ16 stereomicroscope illuminated with a Leica KL1500 LCD light source and a Wild M38 stereomicroscope illuminated with an Applied Scientific Devices Corp. Eco-light 20 fiber optic light source. After sorting, specimens were stored individually or by family in 2 mL microtubes in 70% ethanol until they could be pinned or pointed.

Asilidae were identified by author Barnes, who is an internationally recognized robber fly expert. Specimens of other families were identified using published keys (Table 1).

Asilidae were the focal group of this study; all specimens were removed when bulk samples were sorted so specimens reported here reflect the seasonality and relative abundance of the species sampled by the traps at the site. Specimens of other families were not consistentatly removed by everyone who processed samples, so specimens reported here are indicative of a species presence at the site but not other measurements such as seasonality and relative abundance.

All specimens are deposited in the University of Arkansas Arthropod Museum (UAAM).

## Geographic coverage

### Description

The survey was conducted within a 4 hectare plot established at Steel Creek along the Buffalo National River in Newton County, Arkansas, centered at approximately N 36°02.269', W 93°20.434'. The site is primarily 80–100 year old mature second-growth Eastern mixed deciduous forest dominated by oak (*Quercus*) and hickory (*Carya*), though American beech (*Fagus grandifolia*) and eastern red cedar (*Juniperus virginiana*) are also abundant. A small (14 m x 30 m), fishless pond and glade (10 m x 30 m) with sparse grasses are present within the boundaries of the site. See Skvarla et al. (2015) for additional details.

### Coordinates

36.0367 and 36.0397 Latitude; -93.3917 and -93.3397 Longitude.

## Taxonomic coverage

### Taxa included

**Table:** 

Rank	Scientific Name	
order	Diptera	

## Usage rights

### Use license

Creative Commons CCZero

## Data resources

### Data package title

Steel Creek survey

### Number of data sets

1

### Data set 1.

#### Data set name

Steel Creek Symphyta

#### Data format

Darwin Core Archive

#### Number of columns

34

#### Download URL

http://dx.doi.org/10.5061/dryad.bk225

#### 

**Data set 1.** 

Column label	Column description
typeStatus	Nomenclatural type applied to the record
catalogNumber	Unique within-project and within-lab number applied to the record
recordedBy	Who recorded the record information
individualCount	The number of specimens contained within the record
lifeStage	Life stage of the specimens contained within the record
kingdom	Kingdom name
phylum	Phylum name
class	Class name
order	Order name
family	Family name
genus	Genus name
specificEpithet	Specific epithet
scientificNameAuthorship	Name of the author of the lowest taxon rank included in the record
scientificName	Complete scientific name including author and year
taxonRank	Lowest taxonomic rank of the record
country	Country in which the record was collected
countryCode	Two-letter country code
stateProvince	State in which the record was collected
county	County in which the record was collected
municipality	Closest municipality to where the record was collected
locality	Description of the specific locality where the record was collected
verbatimElevation	Average elevation of the field site in meters
verbatimCoordinates	Approximate center point coordinates of the field site in GPS coordinates
verbatiumLatitude	Approximate center point latitude of the field site in GPS coordinates
verbatimLongitude	Approximate center point longitude of the field site in GPS coordinate
decimalLatitude	Approximate center point latitude of the field site in decimal degrees
decimalLongitude	Approximate center point longitude of the field site in decimal degrees
georeferenceProtocol	Protocol by which the coordinates were taken
identifiedBy	Who identified the record
eventDate	Date or date range the record was collected
habitat	Description of the habitat
language	Two-letter abbreviation of the language in which the data and labels are recorded
institutionCode	Name of the institution where the specimens are deposited
basisofRecord	The specific nature of the record

## Additional information

### Results

We collected and identified specimens representing 12 families, 27 genera, and 38 species during this study (Table 2). Seven species, which represent 18% of the total species identified, are recorded for the first time from Arkansas.

### Notes on newly reported species

*Lasiopogon opaculus* Loew, 1874 (Asilidae) (Fig. 1) is known from Ontario south through Georgia, west to Illinois, Nebraska, and Mississippi (Cannings 2002).

**Figure 2.**
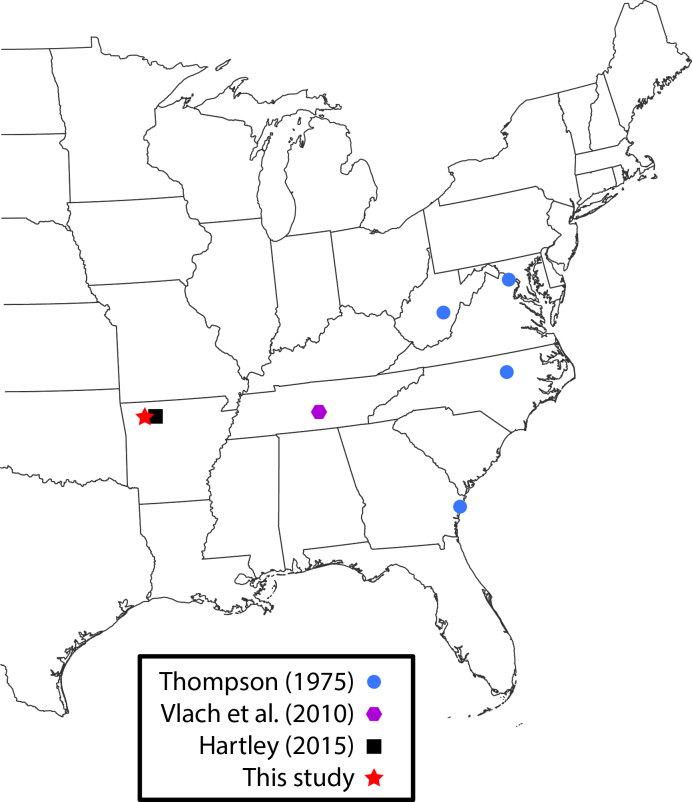
*Lygistorrhina sanctaecatharinae* collection localities.

*Lygistorrhina santaecatharinae* (Lygistorrhinidae) has only been reported in the literature on two occasions and is known from localities in West Virginia, Virginia, Georgia, and Tennessee (Fig. 2) (Thompson 1975, Vlach et al. 2010). Shortly after the specimens reported herein were collected, photographs of female *L. santaecatharinae* feeding on a flower, possibly *Rudbeckia*, from Buffalo Point recreation area (Marion County, Arkansas), a locality 70 km away from Steel Creek, were posted online (Fig. 3, via Hartley 2015). Not only do the photographs provide an additional locality in Arkansas, but they also provide the first record of nectivory in *L. santaecatharinae* and apparently the first record of feeding or indeed any behavior in a lygistorrhinid.

**Figure 4.**
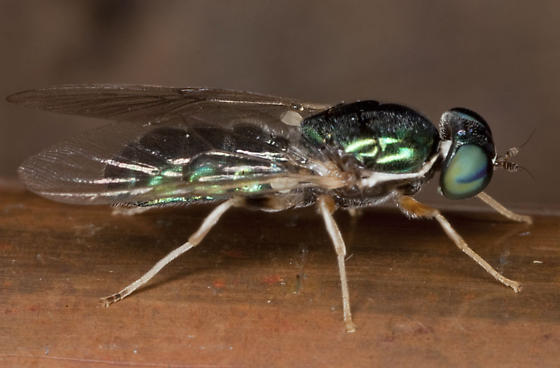
*Cephalochrysa nigricornis*. Photograph by Steve Nanz, used with permission.

*Cephalochrysa nigricornis* (Loew, 1866) (Stratiomyidae) (Fig. 4) is known from Quebec and Ontario south to Georgia, west to Wisconsin, Minnesota, and Kansas (Woodley 2001).

*Gowdeyana punctifera* (Malloch, 1915) (Stratiomyidae) is widespread in eastern North America and occurs from Massachusetts south to Alabama, west to South Dakota, Wyoming, Utah, and Morelos and Sinaloa, Mexico (Woodley 2001).

**Figure 5.**
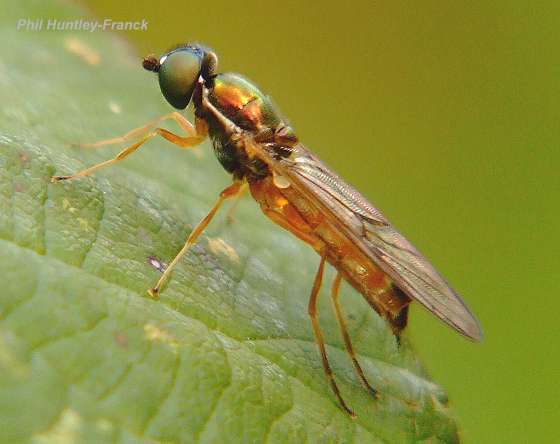
*Sargus decorus*. Photograph by Phil Huntley-Franck, used with permission.

*Sargus decorus* Say, 1824 (Stratiomyidae) (Fig. 5) is widespread in North America and occurs from Quebec and Ontario, south to Georgia, west to Yukon, British Columbia, Washington, and California (Woodley 2001).

*Callopistromyia annulipes* Macquart, 1855 (Ulidiidae) is widespread in North America and has been reported from Maine south to Louisiana, west to Washington (Kameneva and Korneyev 2005).

**Figure 6.**
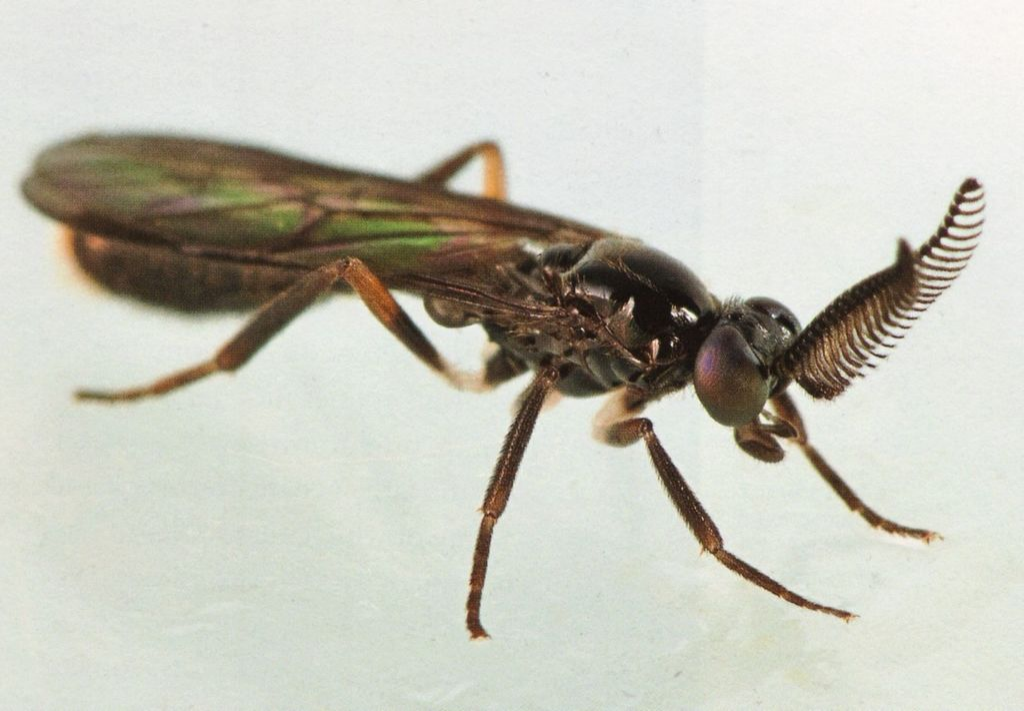
*Rachicerus obscuripennis*. Photograph by Stephen A. Marshall, used with permission

**Figure 7.**
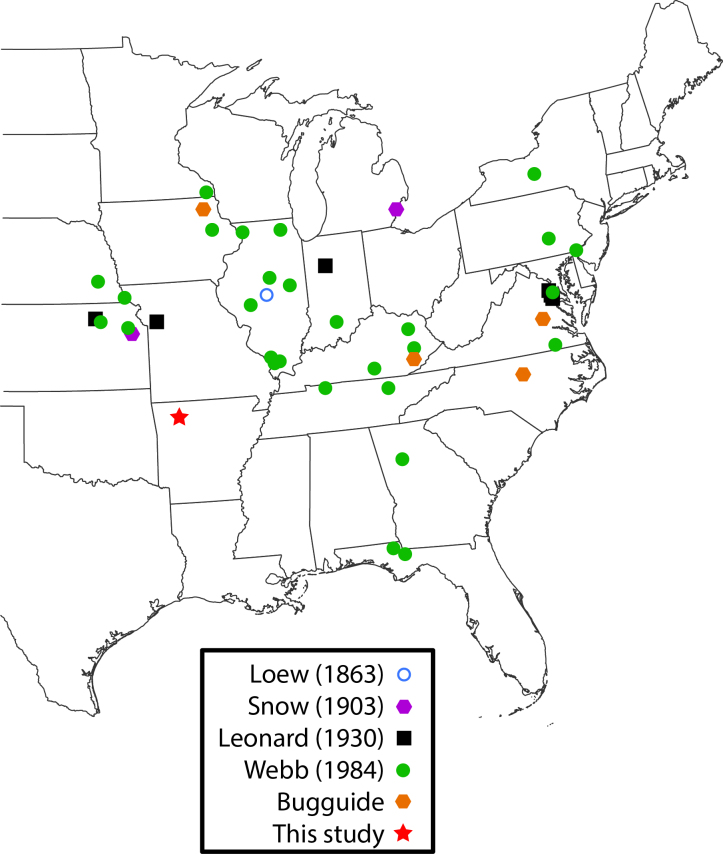
*Rachicerus obscuripennis* collection localities, including those from Loew 1863, Snow 1903, Leonard 1930, Webb 1984; Bugguide localities from Coin 2006, Hatfield 2009, Bentley 2014).

*Rachicerus obscuripennis* Loew, 1863 (Xylophagidae) (Fig. 6) is the only species of the genus in the eastern United States with pectinate antennae; it is found in wooded areas and has been recorded from New York south through Florida, west to Minnesota, Nebraska, Missouri, and Kansas (Fig. 7) (Webb 1984).

### Discussion

It is unsurprising that only one of the twenty species of Asilidae was newly recorded in Arkansas as author Barnes has been studying robber flies in the state for over a decade. However, that such a distinctive species as *Rachicerus obscuripennis* has been known from Missouri since 1901 but is just now reported from neighboring Arkansas illustrates how poorly surveyedsome groups are in the state. This is in line with previous publications in this series, which also reported species previously unrecorded in Arkansas, some of which are quite distinctive (Skvarla et al. 2015, Skvarla et al. 2016b, Skvarla et al. 2016a).

Previous publications have utilized social media and citizen science websites such as Facebook, Flickr, and Bugguide to discover new species (e.g., Winterton et al. 2012, Otto et al. 2014, Gonella et al. 2015) and expand the known range of described species (e.g., Pérez-Hidalgo et al. 2011, Skvarla et al. 2015). The photographs that depict nectivory in *Lygistorrhina sanctaecatharinae* highlight the potential importance of such websites in of the study of natural history and illustrate how they can connect researchers with photographs of behavior in species that are rarely seen alive.

## Figures and Tables

**Figure 1a.**
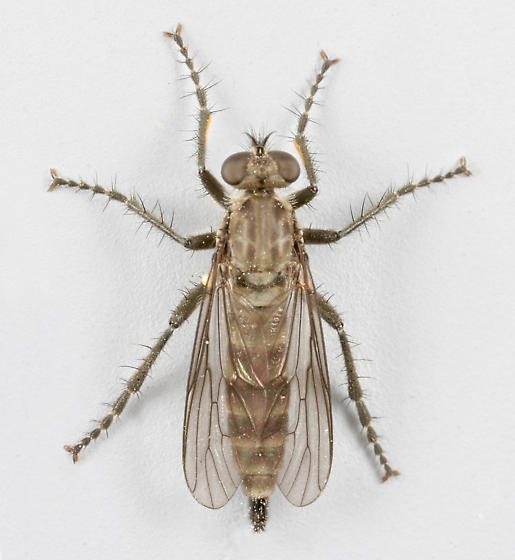
Female, dorsal

**Figure 1b.**
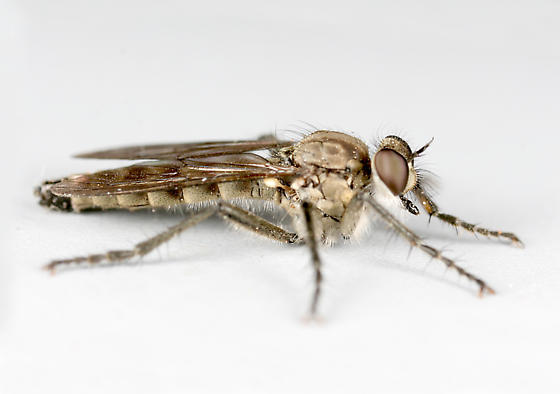
Female, lateral

**Figure 1c.**
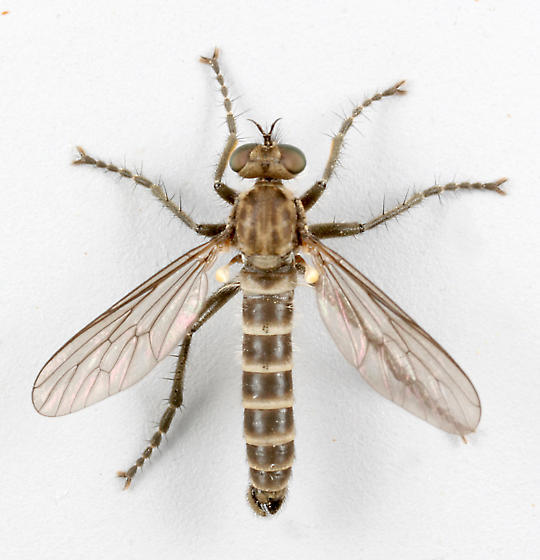
Male, dorsal

**Figure 1d.**
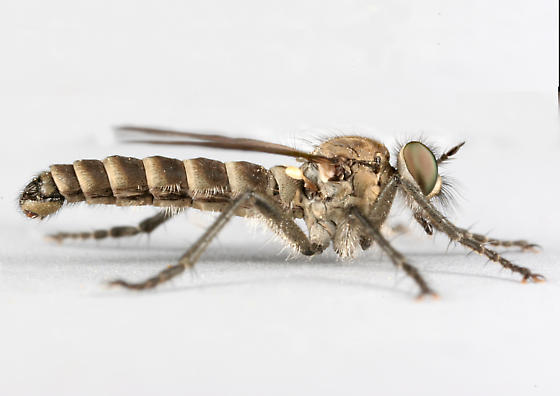
Male, lateral

**Figure 3a.**
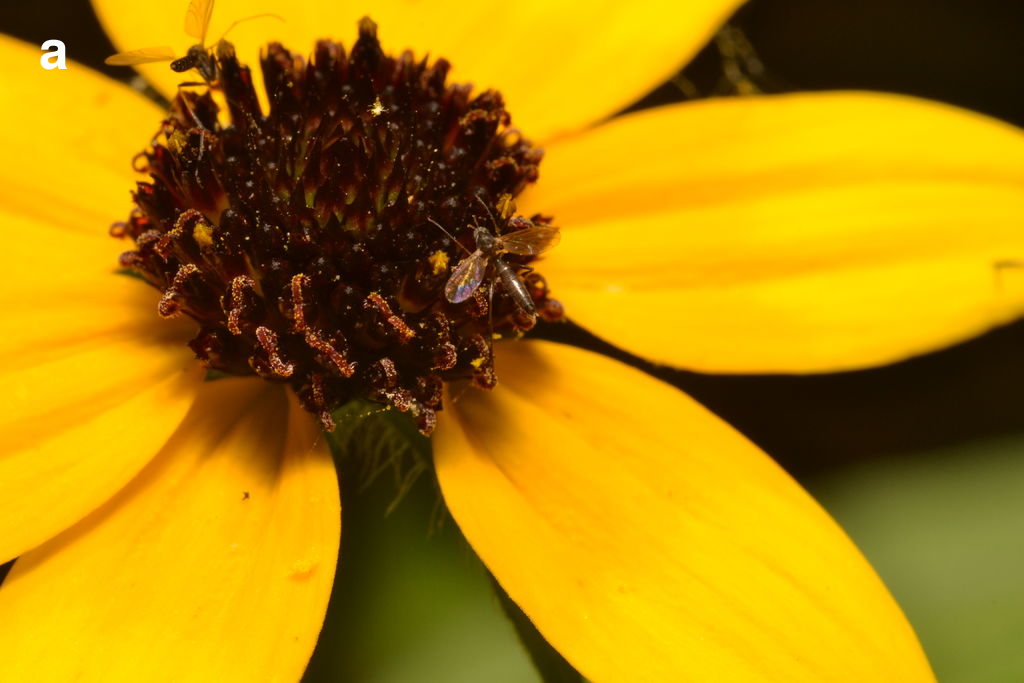
Two *L. sanctaecatharinae* visiting a flower.

**Figure 3b.**
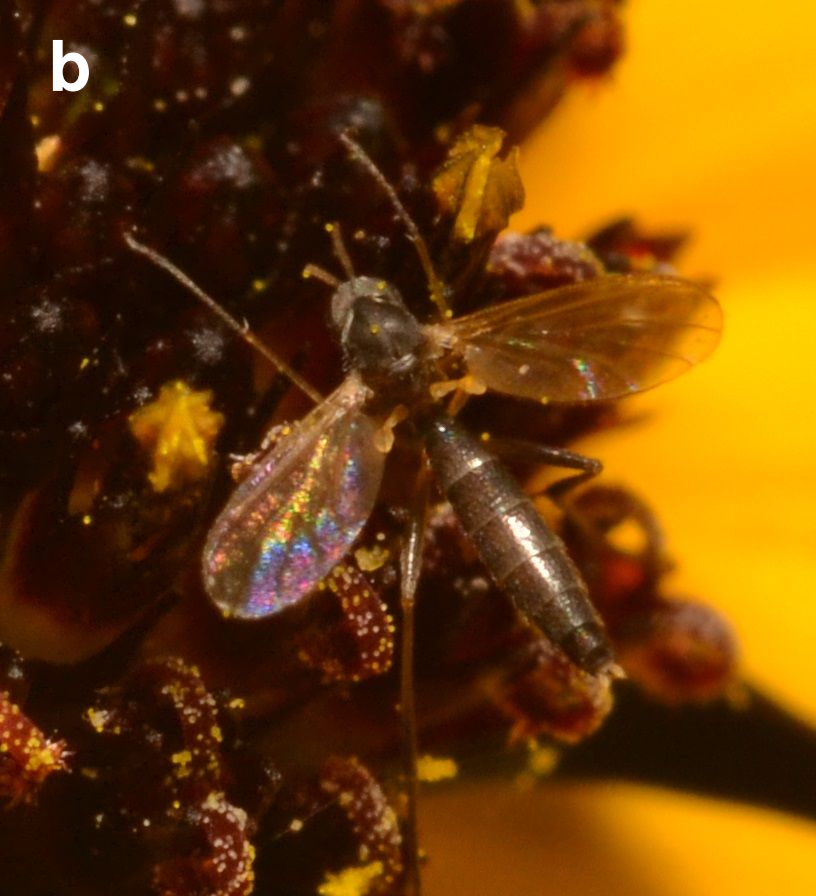
Cropped detail of figure 2a.

**Figure 3c.**
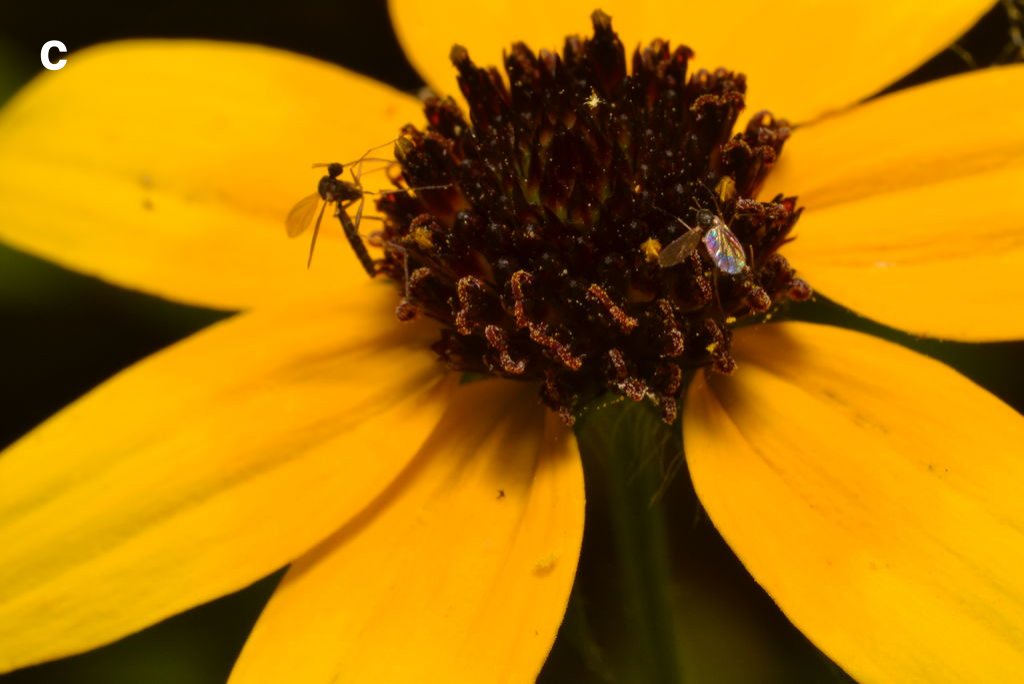
The same two *L. sanctaecatharinae* as figure 2a at slightly different angles.

**Figure 3d.**
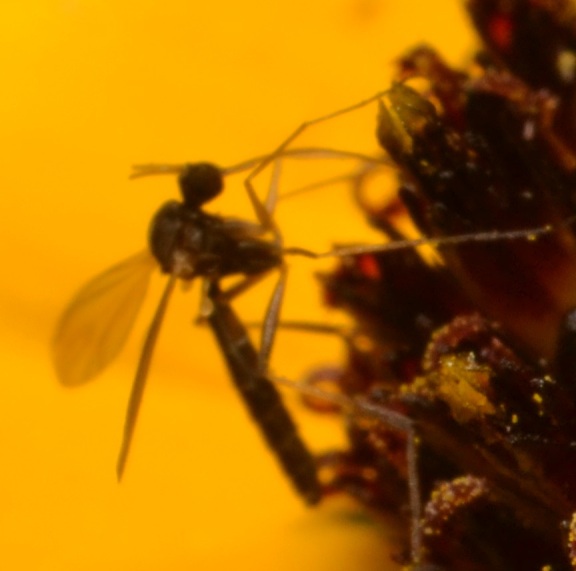
Detail of figure 2c, clearly showing nectivory.

**Table 1.** References used in identification.

**Family**	**Genus**	**Reference**
General identification		McAlpine et al. 1981, McAlpine et al. 1987
Anisopodidae	* Sylvicola *	
Drosophilidae	* Drosophila *	*D. suzukii* is a distinctive species, no key necessary
Drosophilidae	* Zaprionus *	*Z. indianus* is a distinctive non-native species, no key necessary
Lygistorrhinidae	* Lygistorrhina *	Thompson 1975
Mydidae	* Mydas *	*M. clavatus* is a distincitve species in Arkansas, no key necessary
Oestridae	* Cephenemyia *	Bennett and Sabrosky 1972, Taber and Fleenor 2004, Fleenor and Taber 2007
Oestridae	* Cuterebra *	Sabrosky 1986
Ptychopteridae	* Bittacomorpha *	Distinctive genus represented by a single species in the Eastern United States, no key necessary.
Stratiomyiidae		Williston 1885, McFadden 1972, Woodley 2001
Stratiomyiidae	* Ptecticus *	McFadden 1971
Xylophagidae	* Rachicerus *	Webb 1984

**Table 2.** Species collected, including total number of specimens. New state records are indicated by an asterisk (*).

**Family**	**Genus**	**Species**	**Number of specimens**
Anisopodidae	* Sylvicola *	*Sylvicola fenestralis* (Scopoli, 1763)	3
Asilidae	* Diogmites *	*Diogmites misellus* Loew, 1866	6
Asilidae	* Diogmites *	*Diogmites neoternatus* (Bromley, 1951)	1
Asilidae	* Efferia *	*Efferia aestuans* (Linnaeus, 1763)	3
Asilidae	* Holopogon *	*Holopogon phaeonotus* Loew, 1874	3
Asilidae	* Laphria *	*Laphria divisor* (Banks, 1917)	2
Asilidae	* Laphria *	*Laphria flavicollis* Say, 1824	15
Asilidae	* Laphria *	*Laphria index* McAtee, 1919	4
Asilidae	* Laphria *	*Laphria sicula* McAtee, 1919	11
Asilidae	* Lasiopogon *	*Lasiopogon opaculus* Loew, 1874*	2
Asilidae	* Leptogaster *	*Leptogaster aegra* Martin, 1957	1
Asilidae	* Leptogaster *	*Leptogaster brevicornis* Loew, 1872	10
Asilidae	* Leptogaster *	*Leptogaster flavipes* Loew, 1862	1
Asilidae	* Leptogaster *	*Leptogaster virgata* Coquillett, 1904	5
Asilidae	* Machimus *	*Machimus antimachus* (Walker, 1849)	22
Asilidae	* Machimus *	*Machimus sadyates* (Walker, 1849)	6
Asilidae	* Machimus *	*Machimus virginicus* (Banks, 1920)	3
Asilidae	* Neoitamus *	*Neoitamus flavofemoratus* (Hine, 1909)	33
Asilidae	* Ommatius *	*Ommatius gemma* Brimley, 1928	2
Asilidae	* Ommatius *	*Ommatius ouachitensis* Bullington and Lavigne, 1984	3
Asilidae	* Taracticus *	*Taracticus octopunctatus* (Say, 1823)	3
Drosophilidae	* Drosophila *	*Drosophila suzukii* (Matsumura, 1931)	9
Drosophilidae	* Zaprionus *	*Zaprionus indianus* Gupta, 1970	1
Limoniidae	* Cladura *	*Cladura flavoferruginea* Osten Sacken, 1859	26
Lygistorrhinidae	* Lygistorrhina *	*Lygistorrhina sanctaecatharinae* Thompson, 1975*	2
Mydidae	* Mydas *	*Mydas clavatus* (Drury, 1773)	3
Oestridae	* Cephenemyia *		2
Oestridae	* Cuterebra *	*Cuterebra emasculator* Fitch, 1856	1
Oestridae	* Cuterebra *	*Cuterebra f. fontinella* Clark, 1827	4
Ptychopteridae	* Bittacomorpha *	*Bittacomorpha clavipes* (Fabricius, 1781)	1
Stratiomyidae	* Cephalochrysa *	*Cephalochrysa nigricornis* (Loew, 1866)*	1
Stratiomyidae	* Gowdeyana *	*Gowdeyana punctifera* (Malloch, 1915)*	1
Stratiomyidae	* Ptecticus *	*Ptecticus trivattus* (Say, 1829)	680
Stratiomyidae	* Sargus *	*Sargus decorus* Say, 1824*	2
Tipulidae	* Tanyptera *	*Tanyptera dorsalis* (Osten Sacken, 1864)	2
Ulidiidae	* Callopistromyia *	*Callopistromyia annulipes* (Macquart, 1855)*	1
Ulidiidae	* Idana *	*Idana marginata* (Say, 1830)	6
Xylophagidae	* Rachicerus *	*Rachicerus obscuripennis* Loew, 1863*	4
